# Design and characteristics analysis of a new vibration reduction system for in service long span transmission tower

**DOI:** 10.1038/s41598-022-15659-9

**Published:** 2022-07-05

**Authors:** Jinghua Liu, Ziming Li, Wenwu Liu, Changsheng Hu, Chunhua Zhang

**Affiliations:** 1 Equipment Department of State Grid Corporation of China, Beijing, 100031 China; 2 State Grid Heilongjiang Electric Power Co., Ltd., Harbin, 150090 Heilongjiang China; 3 College of Engineering, Northeast Agricultural University, Harbin, 150030 Heilongjiang China; 4 School of Mechatronics Engineering, Harbin Institute of Technology, Harbin, 150001 Heilongjiang China; 5 Dandong Power Supply Company of Liaoning Electric Power Co. Ltd., Dandong, 11800 China

**Keywords:** Electrical and electronic engineering, Energy science and technology, Engineering

## Abstract

The wind-induced fatigue is the main factor leading to reduction of the bearing capacity of long-span transmission towers. In order to reduce the harm of wind vibration, this paper takes the 500 kV Jiamusi region ISLSTT (in service long span transmission tower) as the research object, and a new kind of vibration reduction system is proposed based on a steel wire rope damping structure, with which the vibration characteristics of ISLSTT is analyzed. Firstly, the layout and components of the new vibration reduction system are described, and the damping performance of which is verified and analyzed by finite element method. Secondly, the nonlinear finite element dynamic simulation model of ISLSTT with the new vibration reduction system is established, and the multi-dimensional fluctuating wind speed time history satisfying Davenport wind speed spectrum is given by harmonic superposition method in the time domain. Based on the Bernoulli theorem, the corresponding time history of wind pressure is obtained, and the random wind load is applied to the finite element model to verify the feasibility and efficient of the new vibration reduction system. Finally, the aero-elastic wind tunnel test model of ISLSTT with the new vibration reduction system is built, and the time history curves of stress and acceleration at key points under different wind directions are obtained. By comparing with the un-damped system, it is demonstrated that the average damping efficiency of this method in the scale of stress and acceleration is 72.88% and 77.17%, respectively. The simulation and wind tunnel test results also demonstrate that the vibration reduction system based on wire rope damping structure can effectively reduce the vibration of ISLSTT caused by the non-uniformity of wind speed. The research results lay a solid foundation for the vibration reduction design of in service long span tower-line system in future.

## Introduction

With the development of economy to high quality, the proportion of power construction in the national economy is gradually increasing, and the ISLSTT supporting power transmission is showing a higher and greater trend. ISLSTT is a typical high-rise structure, which is prone to collapse accidents due to its high height and large slenderness ratio. Compare to other loads, wind load is the main inducement of high-rise structure accidents^1,2^ and a large number of studies have shown that wind-induced fatigue is the key factor leading to the reduction of the bearing capacity of high-rise structures^3,4^. Therefore, it has an important engineering significance to design and research the vibration reduction system of ISLSTT.

The fatigue cumulative damage of ISLSTT could be effectively reduced by the structural vibration reduction method which will improve the wind resistance of the tower as well. Li et al.^5^ proposed a method of suspension mass pendulum to reduce the seismic response of structure. Li et al.^6,7^ designed a FPS type MTMD system and studied the damping control of the system under the earthquake of transmission tower line system by using ANSYS software. Combined with the structural characteristics of ordinary rolling ball isolation bearing, Cilsalar et al.^8^ designed and developed a new SMA rolling isolation bearing, which makes up for the defects of traditional bearing and realizes the horizontal isolation of transmission tower. Guo et al.^9^ proposed a novel rotational friction negative stiffness damper composed of a negative stiffness device and rotational friction dampers to develop flexible connection for improving the seismic performance of an asymmetrical super high-rise twin-tower connected structure. Liu et al.^10^ proposed a new type of base rolling isolation device, which can overcome the disadvantage that the rolling body of the traditional isolation bearing may roll out of the rolling plate and it has a good damping effect on the transmission tower. Hao et al.^11^ applied the tuned viscous mass damper for vibration control of adjacent buildings by inter-connecting sub-structures. In order to control the seismic response of large-scale structures, Pardo-Varela et al.^12^ investigates the development of a semi-active piezoelectric friction damper. Eskandari et al.^13^ developed a kriging model for predicting mechanical properties of a visco-elastic damper and applied it to fragility analysis of a case study structure retrofitted with visco-elastic dampers. By putting forward the vibration suppression strategy of transmission tower based on the energy dissipation principle of damper and according to the structural characteristics of tower, Zhong et al.^14^ designed and compared the suppression effects of various damper layout schemes on wind vibration. Battista et al.^15^ designed a nonlinear pendulum damper to suppress the first-order modal vibration of the transmission tower. Park et al.^16^ proposed a damping device based on friction damper, which increases the hysteretic loop area of the system and improves the energy consumption of the structure. Tian et al.^17^ studied the seismic response performance of collision TMD control line structure, and developed bidirectional collision TMD on this basis. Chen et al.^18^ proved that the viscous damper installed on the cross arm can significantly reduce the seismic response of the tower. Chen et al.^19,20^ carried out a poor response control and performance evaluation of tower line system with friction damper under strong earthquake. Based on the working characteristics of piezoelectric ceramics, Zhan et al.^21^ developed a self resetting variable friction damper and proposed different connection forms of the damper. Liu et al.^22^ designed and manufactured an eddy current damping for elastic wind tunnel tests of a solar tower, and the results showed that the wind-induced responses could be obviously reduced.

According to the above analysis, it’s obvious that the vibration of transmission tower can be reduced by using dampers under the action of wind or earthquake load. However, the existing research mainly focuses on the performance of improvement, quantity optimization and layout location selection of a specific damper during the design stage of transmission tower not the in-service stage. Being a random load, the wind load is the main one leading structural vibration, and which can be reduced by increasing the dynamic stiffness of the whole structure. As the main wind protection measure of high-rise beam and rod composite structure, the steel wire rope is widely used in heavy equipment such as the tower crane. The reason is that by means of adjusting the pre-stress and sectional diameters of steel wire rope, the stiffness of the system can be increased and the adverse impact of wind vibration can be reduced.

Therefore, this paper takes the 500 kV ISLSTT in Jiamusi region of Heilongjiang province in China as the research object. A new vibration reduction system based on the steel wire rope damping structure is designed, and the simulated random wind load is applied to the finite element model of the ISLSTT by using the nonlinear finite element method in the time domain. Further, a scale model of wind tunnel test of the ISLSTT with the new vibration reduction system is also build for comparing the structural acceleration and stress response without and with damping structure to verify the feasibility and damping efficiency of this vibration reduction system.

## Vibration reduction scheme of steel wire rope damping structure

### Structure of vibration reduction system

**Figure 1:**
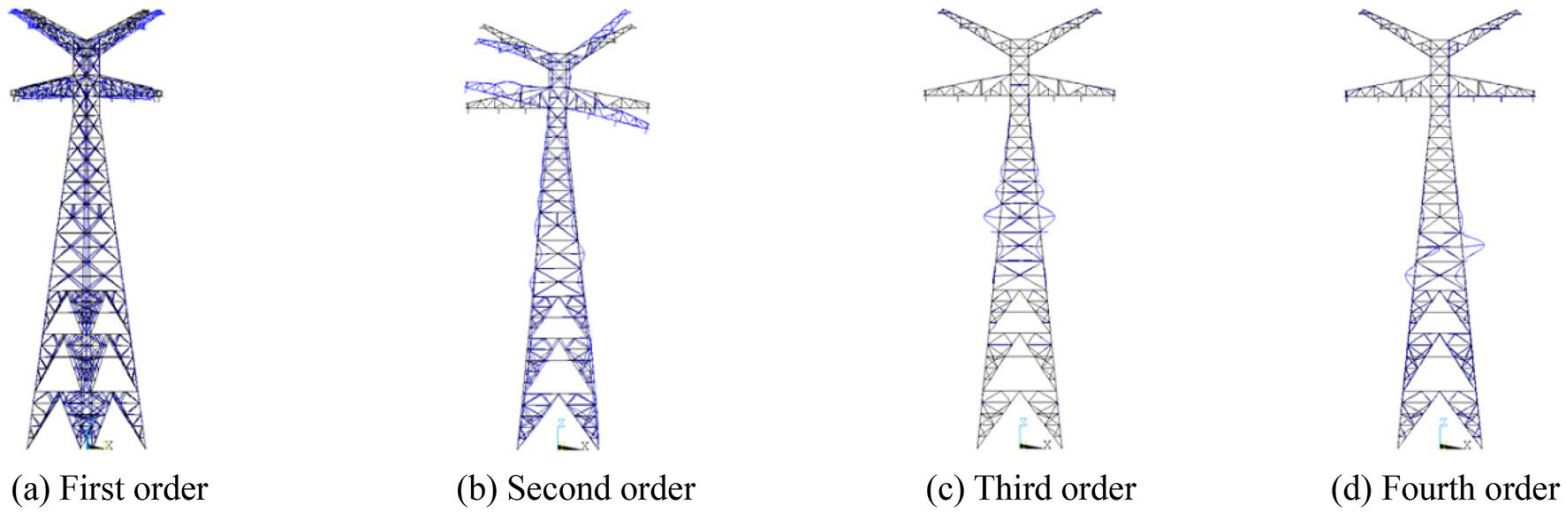
Modal modes of 500 kV ISLSTT.

The first 4 natural modal modes of the 500 kV ISLSTT are shown in Fig. 1, and their natural frequencies are 3.357 Hz, 3.759 Hz, 4.179 Hz, 4.527 Hz, respectively. According to the modal analysis, the lateral and torsional vibration modes of the 500 kV ISLSTT are large, which indicate that these two modal modes are easily influenced by wind. Therefore, the resistance system based on the steel wire rope is introduced to reduce the lateral and torsional vibration of the 500 kV ISLSTT.

**Figure 2:**
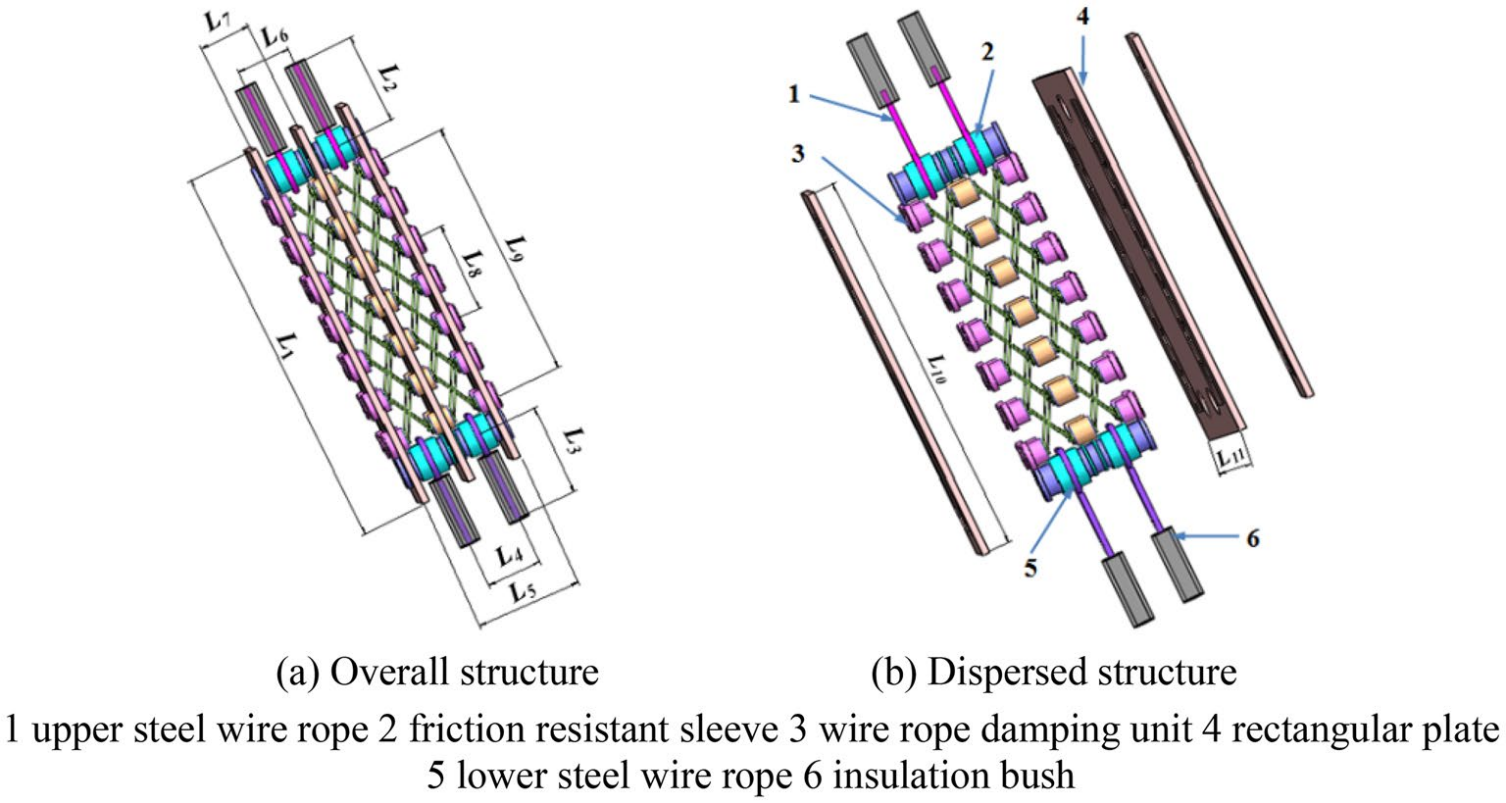
3D structure of vibration reduction system.

In order to reduce the harm of wind vibration to the ISLSTT, and to minimize the structural changes to the transmission tower. The 500 kV ISLSTT with a new vibration reduction system based on a steel wire rope damping structure is taken as the research object in this paper as shown in Fig. 2. The structural dimensions of the vibration reduction system are as following: length of vibration reduction system *L*_*1*_ is 41.6 m, length of upper steel wire rope *L*_*2*_ is 12.6 m, length of lower steel wire rope *L*_*3*_ is 14.5 m, distance between lower steel wire ropes *L*_*4*_ is 2.1 m, distance between outer rectangular plates *L*_*5*_ is 4.0 m, distance between upper steel wire ropes *L*_*6*_ is 2.0 m, distance between inner rectangular plates *L*_*7*_ is 2.0 m, distance between inner damping units *L*_*8*_ is 3.1 m, distance between outer damping units *L*_*9*_ is 9.3 m, length of rectangular plate *L*_*10*_ is 14.5 m and width of rectangular plate *L*_*11*_ is 3.4 m.

**Figure 3:**
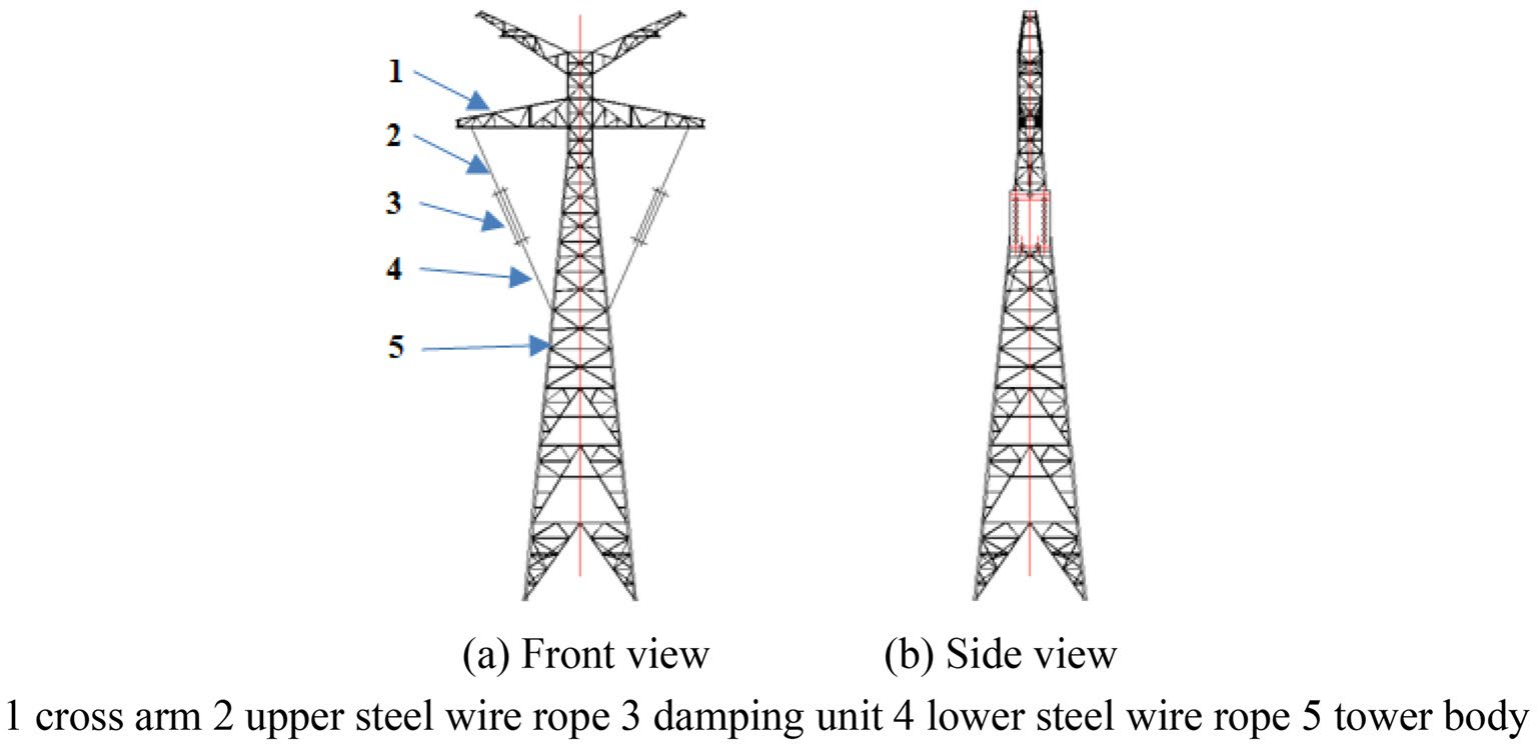
Layout of steel wire rope vibration reduction system.

As shown in Fig. 3, the vibration reduction system is connected to the cross arm and the tower body with steel wire ropes on the left and right sides of the 500 kV ISLSTT, respectively. The design process of the wire rope vibration reduction system is that the damping unit is assembled first, then fixedly connect the damping unit to the cross arm and tower body through the upper and lower wire ropes, and finally adjust the preload in the upper and lower wire ropes by using a threaded connection device. In addition, the outer surface of the upper steel wire ropes and the lower steel wire ropes are wrapped by an insulation bush shown in Fig. 2, which aims to prevent the steel wire rope from contacting with the conductor under the cross arm. The vibration reduction system on both sides of the ISLSTT can increase the composite dynamic stiffness between the cross arm and tower body. At the same time, a rigid flexible coupling space can be formed between the cross arm and the tower body in the condition of high wind speed, which could avoid the fatigue damage of pulsating wind to the main materials of both the tower body and the cross arm.

The steel wire rope vibration reduction system consists of three rectangular plates which are cross connected through the middle wire rope damping unit to form a flexible friction damping damper. It can isolate vibration in three directions and dissipate energy by using the hysteresis damping generated by the flexible friction between each strand of steel wire rope. The damping principle of the new vibration reduction system is to add an elastic damping frame on the outside of the tower body. When the tower body is laterally displaced under the action of wind excitation, the steel wire rope will be further stretched, and the slight elongation of which will keep the lateral displacement of the tower body. Moreover, for avoiding resonance, the tension of the steel wire rope will change when the wind changes, so that the excitation frequency of the wind cannot be consistent with the natural frequency of the tower body.

### Damping performance simulation

**Figure 4:**
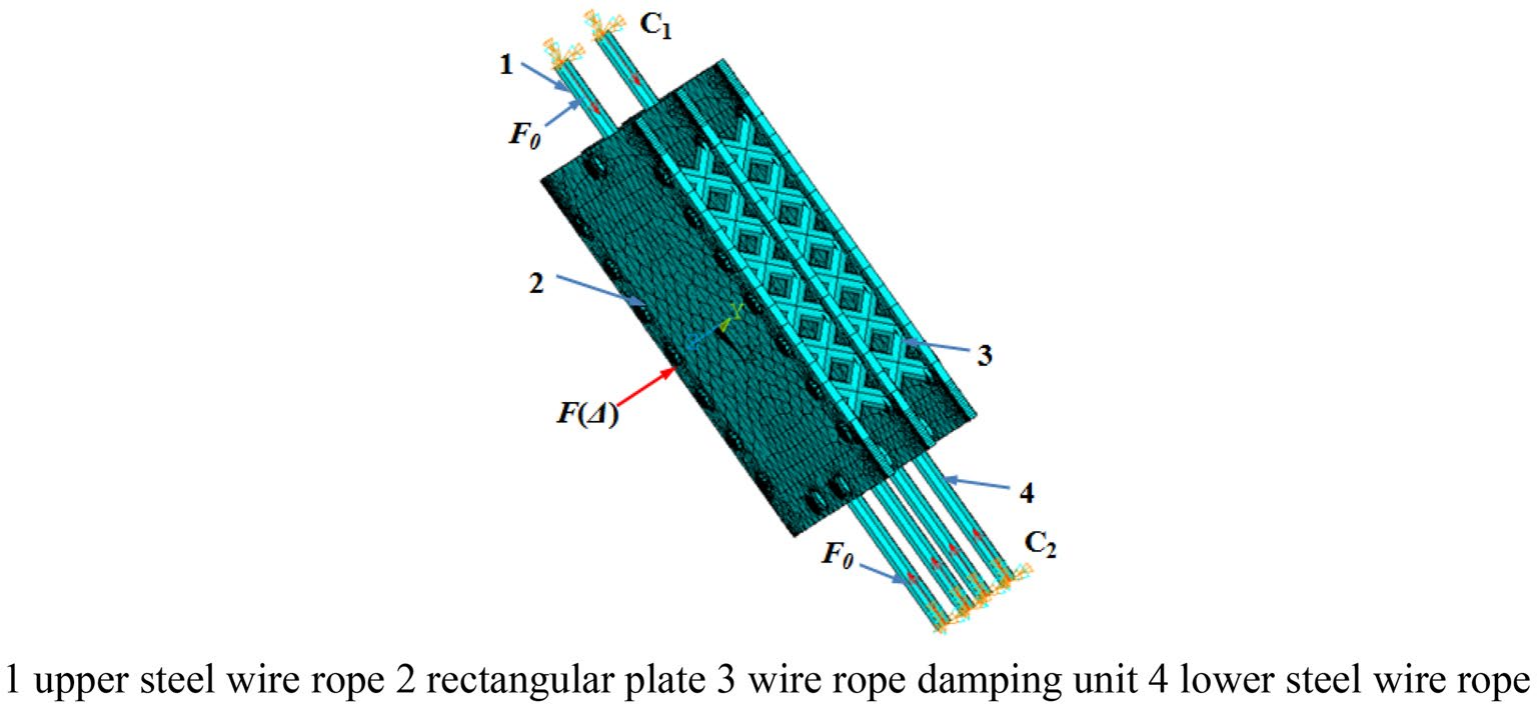
Finite element model of vibration reduction system.

In order to describe the damping performance of the vibration reduction system, the finite element model of which was established as shown in Fig. 4. The materials of the upper and lower wire ropes, rectangular plates and wire rope damping unit in the wire rope damping system are Q345. The diameter *d*_1_ of the wire rope is 30 mm, the thickness *h* of the rectangular plate is 20 mm, and the diameter *d*_2_ of the wire rope in the wire rope damping unit is 25 mm. Constrain the six degrees of freedom at the end of the upper wire rope *C*_1_ and the end of the lower wire rope *C*_2_, respectively. The dynamic load *F*(Δ) is applied perpendicular to the rectangular plates of the wire rope damping system and the displacement in the corresponding direction is obtained. *F*(*Δ*) is the reciprocating displacement load, which size is (1, − 1, 2, − 2, 3, − 3, 4, − 4, 5, − 5, 6, − 6) *Δ*, and *Δ* is the yield displacement of the steel wire rope, which taken as 7.20 mm referring to the steel cylindrical structure. *F*_0_ is the preload applied along the axis of the wire rope in Fig. 4. Shell181 element is used to simulate rectangular plates, link10 element is used to simulate steel wire rope and steel wire rope damping unit. There are 15,570 elements in the finite element model of the wire rope damping system, including 12,324 shell181 elements and 3246 link10 elements. In addition, the degree of freedom coupling method is used to simulate the connection between structures.

**Figure 5:**
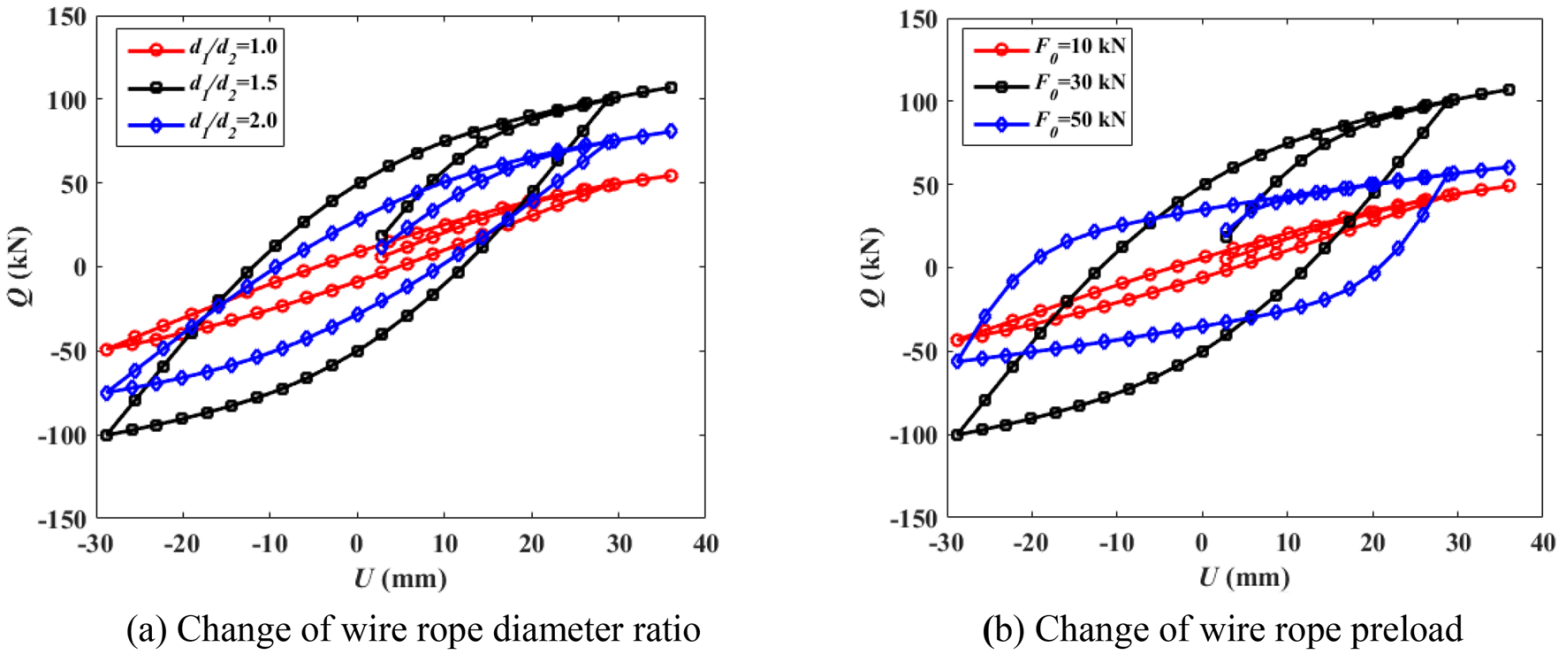
Damping performance of vibration reduction system.

The force to displacement curve of the vibration reduction system under the cyclic action of force *F*(Δ) is obtained in Fig. 5, which can reflect the deformation characteristics, stiffness degradation and energy consumption of the structure in the process of repeated stress, which is the basis for determining the restoring force model and nonlinear dynamic response analysis. The ordinate *Q* represents the restoring force, and the abscissa *U* represents the displacement in the direction of force action.

It can be seen from Fig. 5a that when the preload is constant, the load displacement curve gradually becomes full with the increase of the wire rope diameter ratio, but when the ratio exceeds 1.5, the area enclosed by the load displacement curve decreases and the shape tends to bow, indicating that the energy dissipation capacity of the vibration reduction system decreases. It can be seen from Fig. 5b that when the diameter ratio of steel wire rope is constant, the load displacement curve gradually becomes full with the increase of preload. However, when the preload exceeds 30 kN, the shape of load displacement curve tends to S-shape and the surrounding area decreases, which indicates that the energy dissipation capacity of vibration reduction system decreases.

It can also be seen from Fig. 5 that when the wire rope diameter ratio equals 1.5, and the preload equals 30 kN at the same time, the shape of force to displacement curve of the new vibration reduction system proposed in this paper is similar to the shuttle, which indicates that the plastic deformation capacity of the whole structure is very strong, and has well anti vibration performance and energy dissipation capacity. Therefore, the diameter ratio of steel wire rope is 1.5 and the preload is 30 kN in this paper.

## Nonlinear finite element simulation of 500 kV ISLSTT with the new vibration reduction system

### Nonlinear finite element model

**Figure 6:**
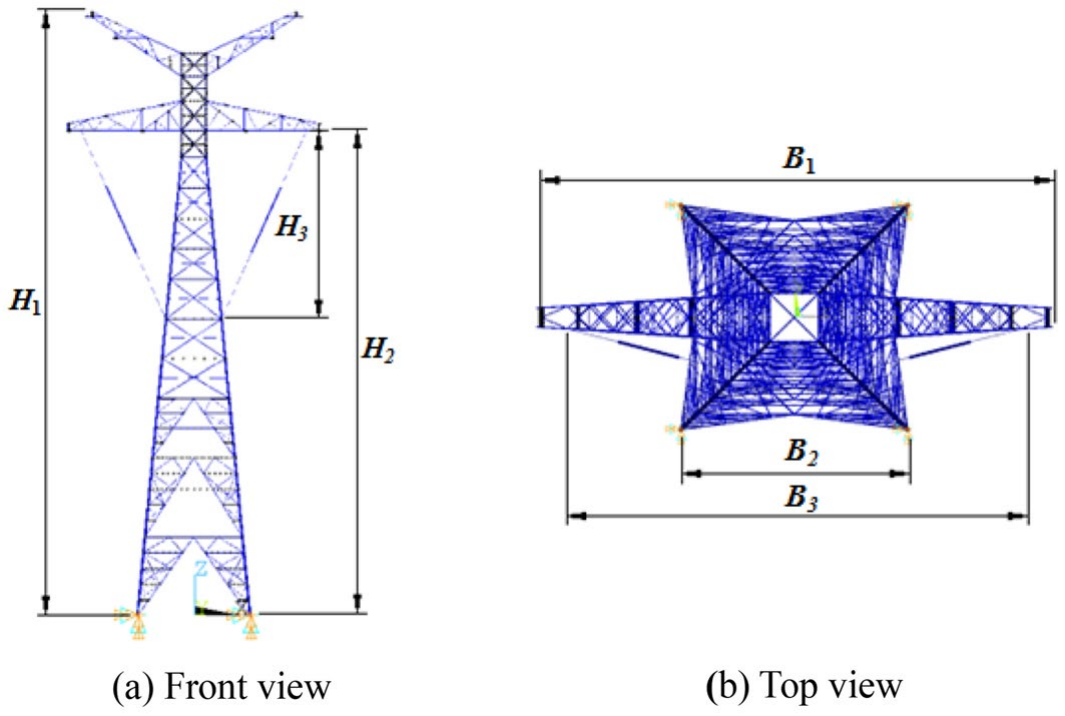
Finite element model of 500 kV ISLSTT with vibration reduction system.

The nonlinear finite element model of 500 kV ISLSTT with the new vibration reduction system for dynamic simulation is established in the finite element software ANSYS as shown in Fig. 6. According to the geological and meteorological data of Jiamusi region of Heilongjiang province in China, the design reference wind speed is 27 m/s, and the ground roughness index is 0.12. The structural parameters values are as following: height of 500 kV ISLSTT *H*_*1*_ is 122.0 m, height of cross arm *H*_*2*_ is 97.0 m, height of vibration reduction system *H*_*3*_ is 35.0 m, width of 500 kV ISLSTT *B*_*1*_ is 51.0 m, width of main chord *B*_*2*_ is 23.0 m, width of vibration reduction system *B*_*3*_ is 45.0 m.

The constraint condition is that it has imposed full constraints of 6 degrees of freedom at the four fulcrums at the bottom of the 500 kV ISLSTT. Since the main chord, diagonal web and transverse web of the long-span steel pipe tower are steel pipe structures, beam188 element is selected for the finite element model of steel pipe, link10 element is selected for the steel wire rope, shell181 element is selected for the rectangular plate, and Q345 steel is used for material of all steel structures.

### Simulation of random wind load

In order to simulate the wind load on the 500 kV ISLSTT, it is assumed that the wind speed $v(z,\;t)$ at any height is a variable with time, which is composed of average wind speed and fluctuating wind speed, that is:

1 $$v(z,\;t) = \overline{v}(z) + v_{d} (z,\;t)$$

where $\overline{v}(z)$ is the average wind speed at height Z can be expressed as follows by Davenport's formula: $\overline{v}(z) = \overline{v}_{10} (z/10)^{\alpha }$, in which $\overline{v}_{10}$ is the average wind speed at the height of 10 m, *α* is the ground roughness index; $v_{d} (z,\;t)$ is the fluctuating wind speed at height Z.

Taking Davenport spectral function as the power spectral density function of fluctuating wind speed at any point in space, there are:

2 $$S(f) = 4K\overline{v}_{10}^{2} \frac{{x^{2} }}{{f(1 + x^{2} )^{4/3} }}$$

In which, $x = 1200\;f/\overline{v}_{10}$, where *f* is the frequency of wind speed, (Hz); *K* is the ground roughness coefficient.

Considering the spatial correlation of fluctuating wind speed, and according to the random process theory, the power spectral cross density function of fluctuating wind speed at any point *i* and *j* in space is:

3 $$S_{i,j} (f) = \sqrt {S_{i,i} (f) \cdot S_{j,j} (f)} coh(f)$$

where the exponential form given by Davenport is:

$$coh(f) = \exp \left[ { - \frac{{2f\sqrt {c_{y}^{2} (y_{1} - y_{2} )^{2} + c_{z}^{2} (z_{1} - z_{2} )^{2} } }}{{\overline{v}(z_{1} ) + \overline{v}(z_{2} )}}} \right]$$

where $S_{n,n} (f)$ is the power spectral density function of fluctuating wind speed at *n* points in space, *n* = *i*, *j*; $coh(f)$ is the square root of coherence function; *c*_*y*_ and *c*_*z*_ are attenuation coefficients of orthogonal direction in any two-point plane; *y*_*i*_ and *z*_*i*_ are coordinates of point *i* in plane, *i* = 1, 2.

According to Bernoulli's theorem, the wind load on any point in space can be expressed by wind pressure *P*(*t*), as shown in formula (4):

4 $$P(t) = \frac{\gamma }{2g}\mu_{s} (z)A(z)\left[ {\overline{v}(z) + v_{d} (z,t)} \right]^{2}$$

where *γ* is the air bulk density; *g* is the gravitational acceleration; $\mu_{s} (z)$ is the shape coefficient at height Z; $A(z)$ is the effective windward area at height Z.

The spectral density matrix *S*(*ω*) of the 500 kV ISLSTT structure under random wind load can be obtained by combining formula (2) and formula (3).

Assuming that there are *n* load points on the structure, and then the wind speed at any point in the space can be obtained by the harmonic superposition method of multidimensional stationary random process:

5 $$v_{d,j} (z,t) = 2\sqrt {\Delta \omega } \sum\limits_{m = 1}^{j} {\sum\limits_{l = 1}^{N} {\left| {H_{j,m} (\omega_{m,l} )} \right|} } \cos (\omega_{m,l} t + \phi_{m,l} )$$

where,

$$\Delta \omega = (\omega_{u} - \omega_{d} )/N,\quad \omega_{m,l} = \omega_{d} + (l - 1)\Delta \omega + \frac{m}{n}\Delta \omega ,\quad (l = 1,\;2, \ldots ,\;N),$$

$H_{j,m} (\omega_{m,l} )$ is the spectral density matrix, then Cholesky decomposition method is used, that is ${\mathbf{S}}(\omega ) = {\mathbf{H}}(\omega ) \cdot {\mathbf{H}}^{*} (\omega )$. Where *N* is the sufficiently large positive integer; $\phi_{m,l}$ is the random phase angle uniformly distributed in [0, 2π); $\omega_{u}$ and $\omega_{d}$ are upper and lower limits of frequency band.

### Simulation analysis of the wind-induced vibration response

**Figure 7:**
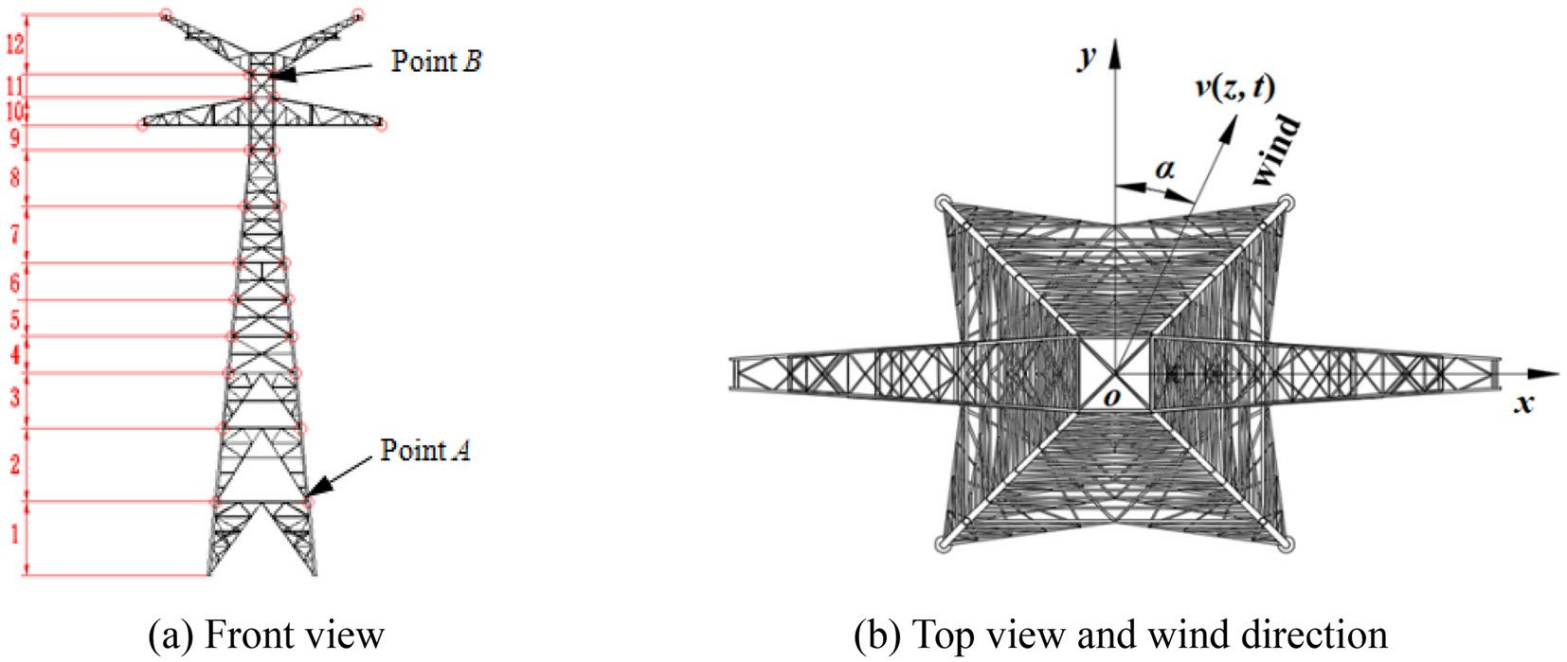
Sectional diagram of ISLSTT and wind pressure action point.

**Figure 8:**
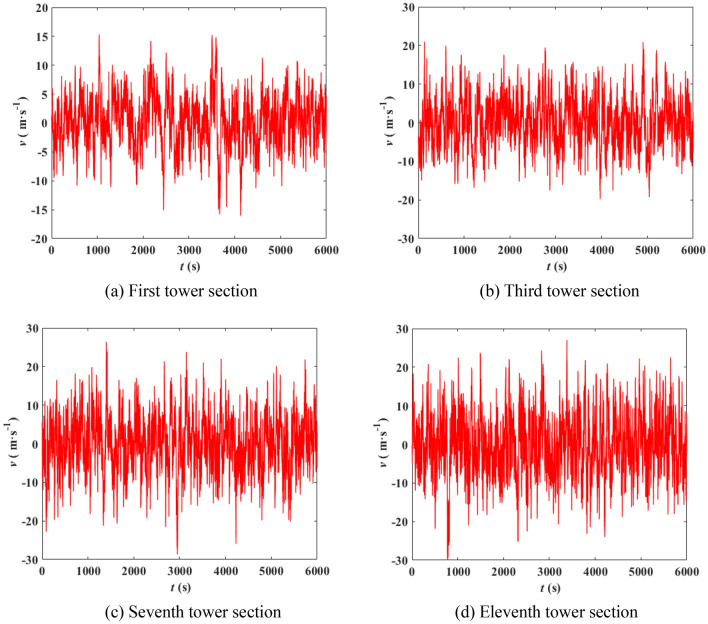
Time history simulation of fluctuating wind speed in typical section of ISLSTT.

**Figure 9:**
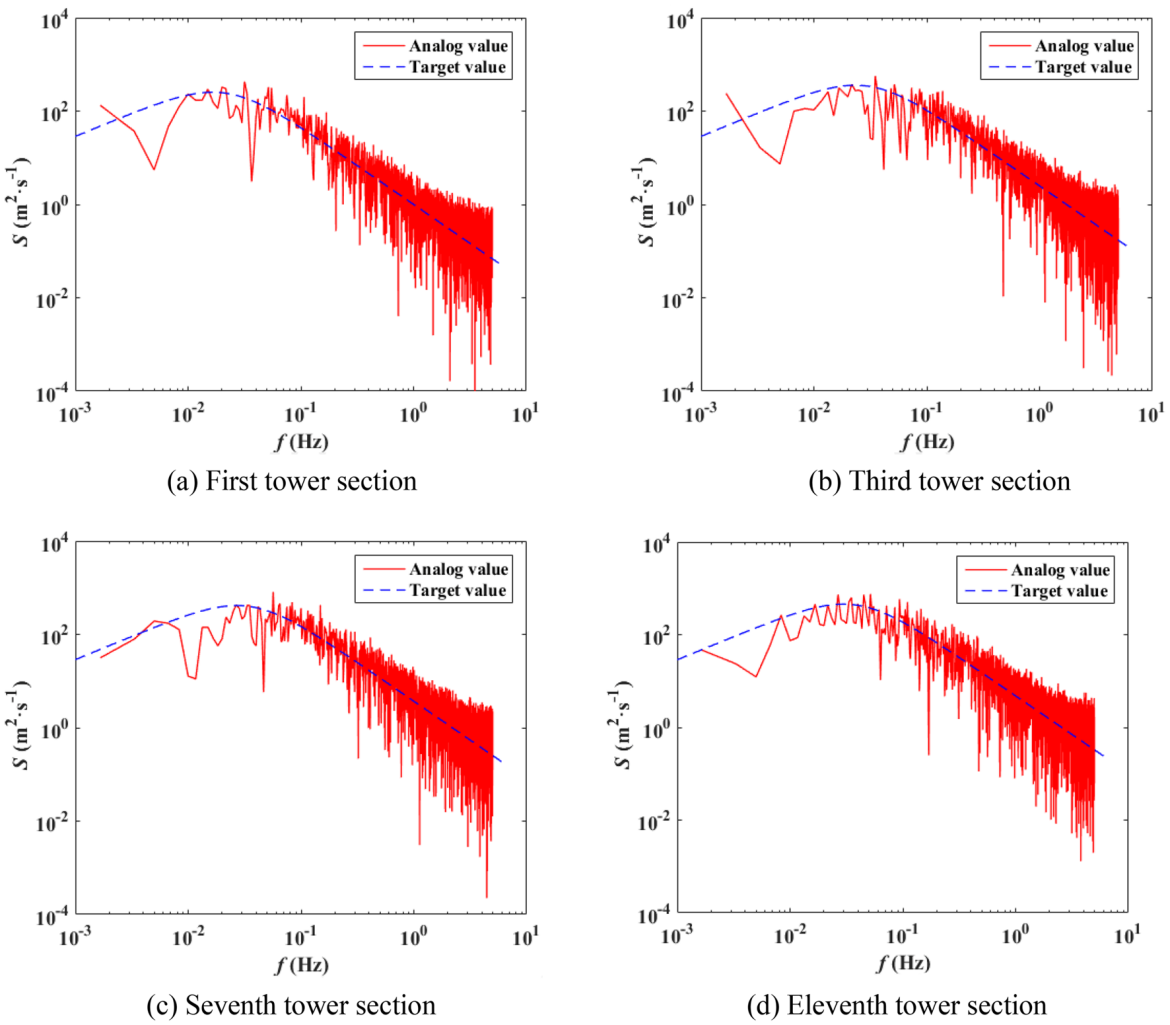
Comparison between simulated wind speed power spectrum and target power spectrum.

The wind pressure on the tower is calculated according to Eq. (4), the 500 kV ISLSTT is treated in sections in this paper, and it is assumed that the wind pressure acts on the discrete nodes shown in Fig. 7a. The magnitude and direction of wind speed are *v*(*z*, *t*) and *α* as shown in Fig. 7b, in which the *y* direction is the direction of connection of conductor and ground wire. Considering the natural mode of the steel pipe tower, when simulating the wind pressure time history, the time step is 0.5 s, and the total time length is 6000 s. When the basic wind speed is 27 m/s, the first tower section, third tower section and the fluctuating wind speed time history curves of seventh tower section and eleventh tower section are shown in Fig. 8. The center heights of each section are 8 m, 38 m, 74.1 m and 106.5 m, respectively. The correctness of each section fluctuating wind speed simulated in this paper is verified by comparing the simulated wind speed power spectrum with Davenport power spectrum. The comparison results are shown in Fig. 9.

**Figure 10:**
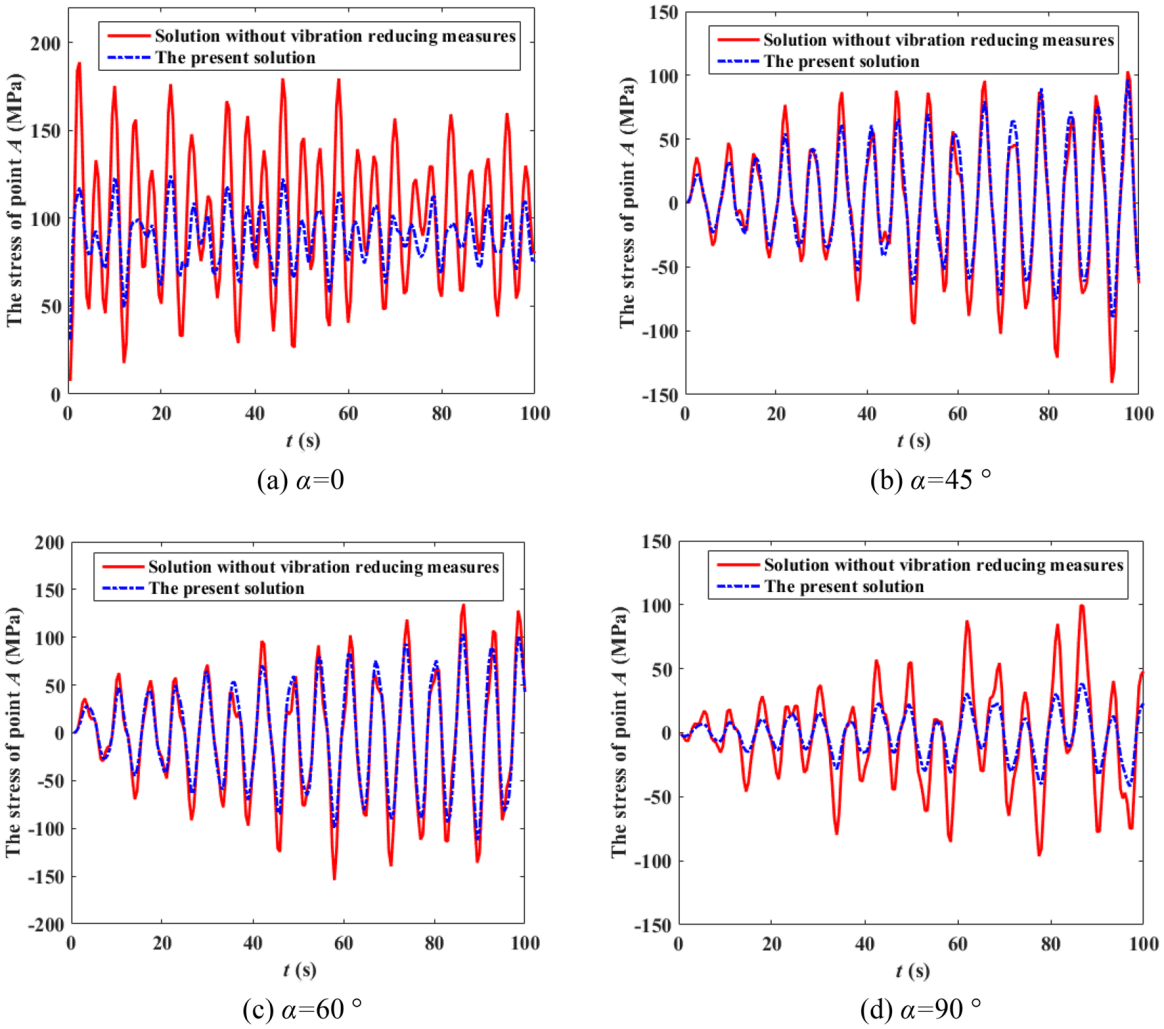
Stress time history curve of point *A* under different wind direction.

**Figure 11:**
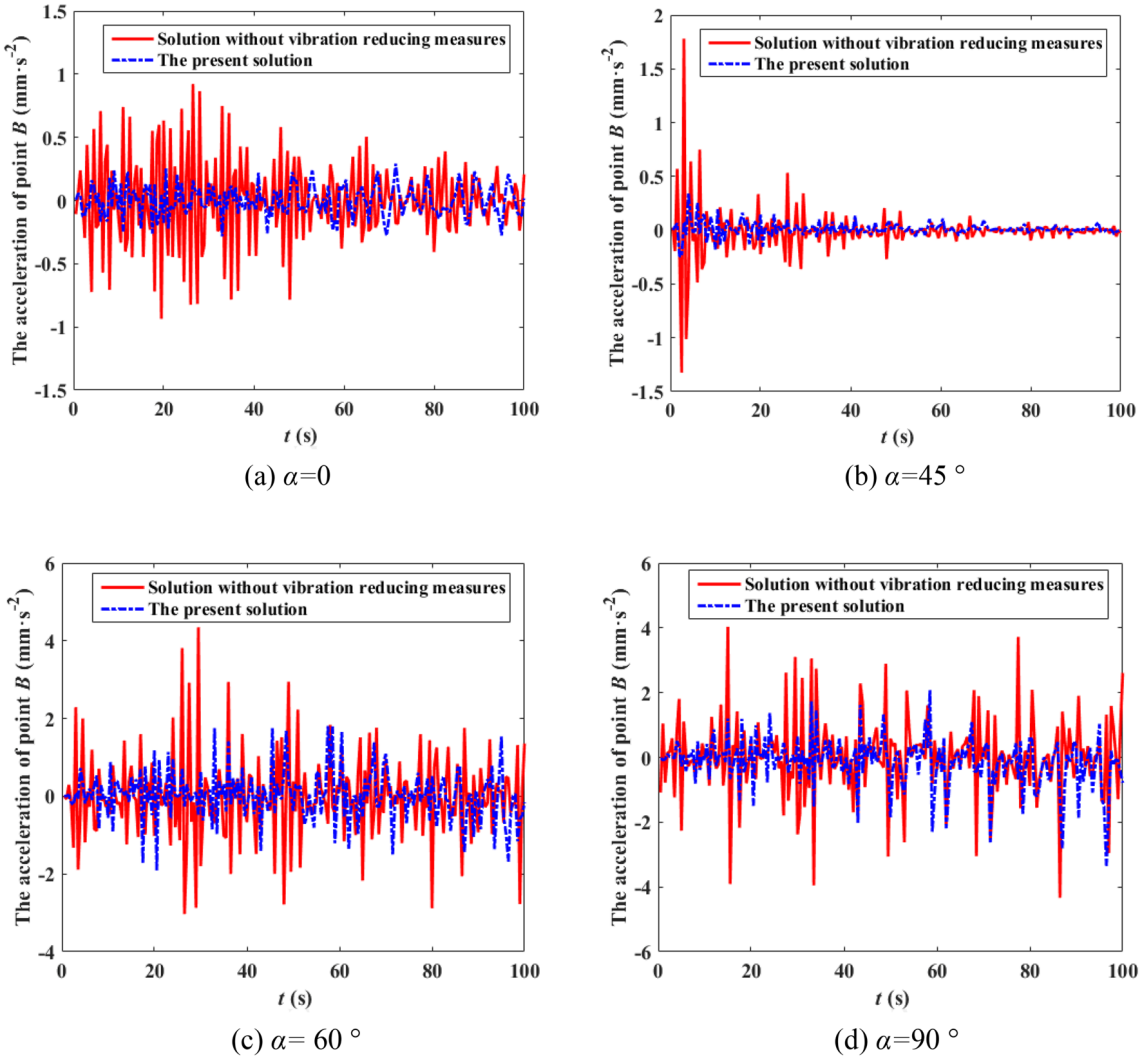
Acceleration time history curve of point *B* under different wind direction.

It can be seen from Fig. 8 that the speed of the wind at various heights are not only different in amplitude, but also in phase, and the correlation of different wind speeds decreases with the increase of distance. It can also be observed from Fig. 9 that the simulated power spectrum in each section of the 500 kV ISLSTT is very consistent with the target power spectrum, which demonstrates the correctness of the fluctuating wind speed established in the paper. By applying the simulated fluctuating wind speed to the nonlinear finite element model of the 500 kV ISLSTT with vibration reduction system shown in Fig. 7, and considering the geometric nonlinearity of the structure, the four fluctuating wind speed direction *α* equals to 0, 45°, 60° and 90° are solved through transient dynamic analysis. Consequently, the stress time history curve of the root point *A* and the acceleration time history curve of the top point *B* of the 500 kV ISLSTT are obtained in Figs. 10 and 11, respectively.

It can be seen from Figs. 10 and 11 that compared with the 500 kV ISLSTT without any vibration reduction system, the new vibration reduction system based on wire rope damping structure established in this paper can significantly reduce the stress of root point *A* under the action of basic wind speed of 27 m/s and wind speed direction *α* equals to 0, 45°, 60° and 90°, and the average vibration reduction efficiency is 66.67%, 65.32%, 61.27% and 68.23%, respectively. The acceleration of the top point *B* is also significantly reduced, and the average damping efficiency is 77.17%, 75.42%, 73.83% and 71.65%, respectively.

## Wind tunnel test of 500 kV ISLSTT with vibration reduction system

### Establishment of aero-elastic test model

**Figure 12:**
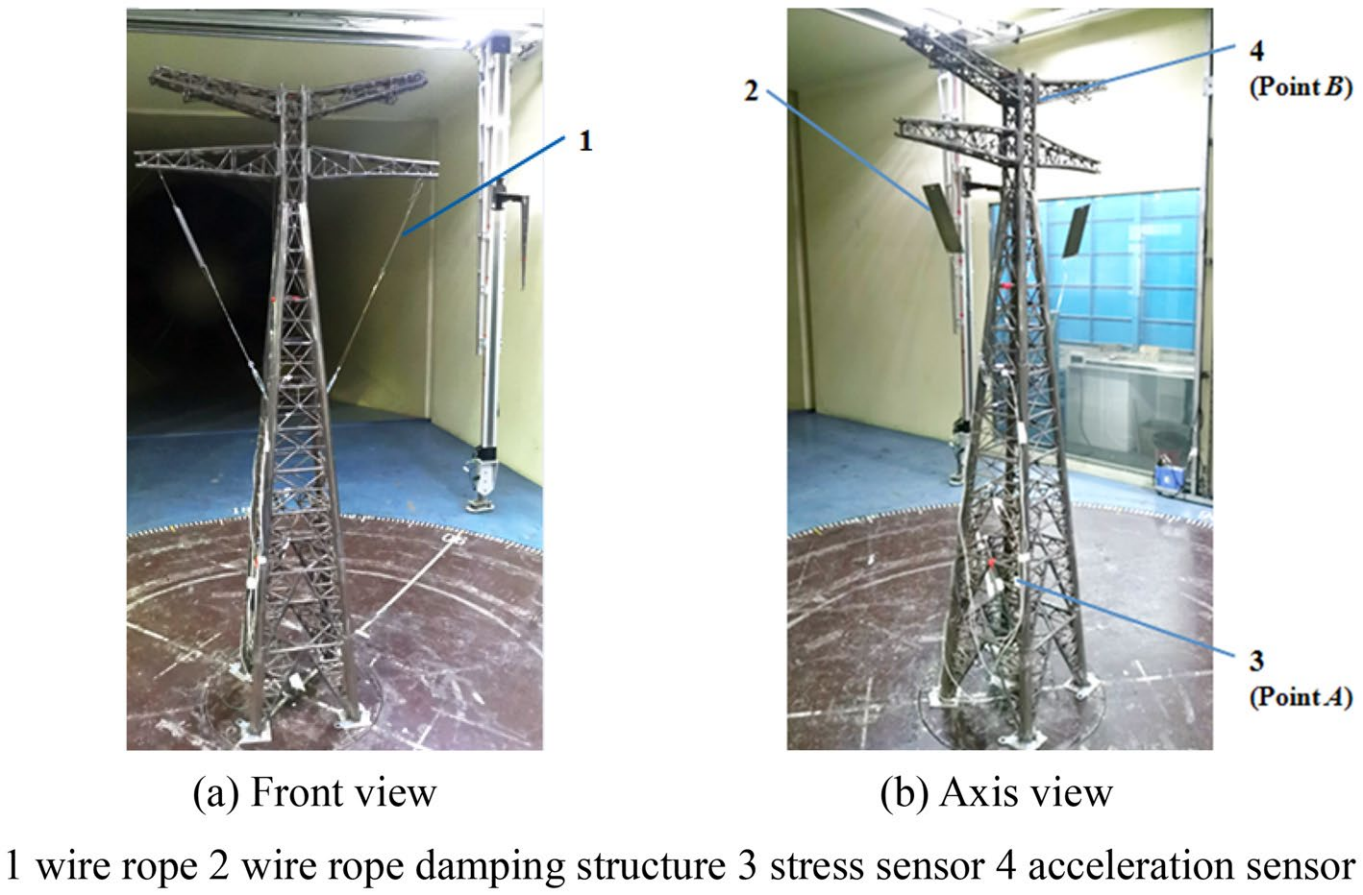
Wind tunnel aero-elastic test model of 500 kV ISLSTT with the new vibration reduction system.

The aero-elastic model of the 500 kV ISLSTT with vibration reduction system is made by discrete stiffness method, in which each member of the transmission tower is regarded as a two force rod according to the mechanical characteristics of the structure, and then the stiffness simulation can meet the similarity of tensile stiffness of members. The similarity coefficients of the 500 kV ISLSTT aero-elastic test model are as following: geometric length *C*_*L*_ is 1/60, geometric area *C*_*A*_ is 1/60^2^, air density *C*_*ρf*_ is 1, structural density *C*_*ρs*_ is 1.8, mass *C*_*m*_ is 1.8/60^3^, tensile stiffness *C*_*EA*_ is 1/3600, frequency *C*_*f*_ is 75.76 and wind speed *C*_*V*_ is 1/2.64. According to above conditions, the wind tunnel aero-elastic test model of the 500 kV ISLSTT with the new vibration reduction system is built as shown in Fig. 12.

In order to facilitate data collection and comparison, the stress sensor is arranged at the root point *A* and the acceleration sensor is arranged at the top point *B* as shown in Fig. 12 above.

### Analysis of wind tunnel test

**Figure 13:**
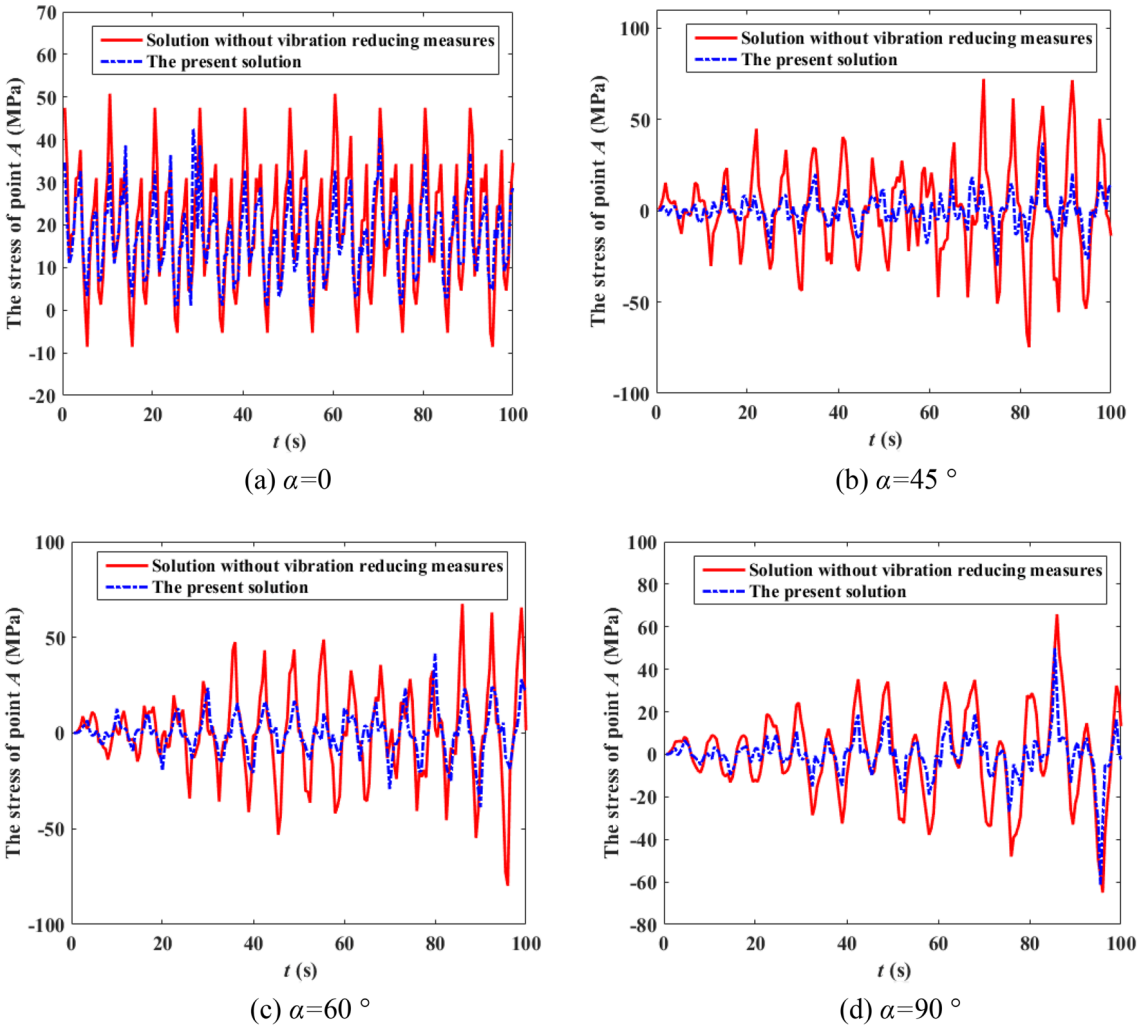
Stress time history curve of point *A* under different wind direction.

**Figure 14:**
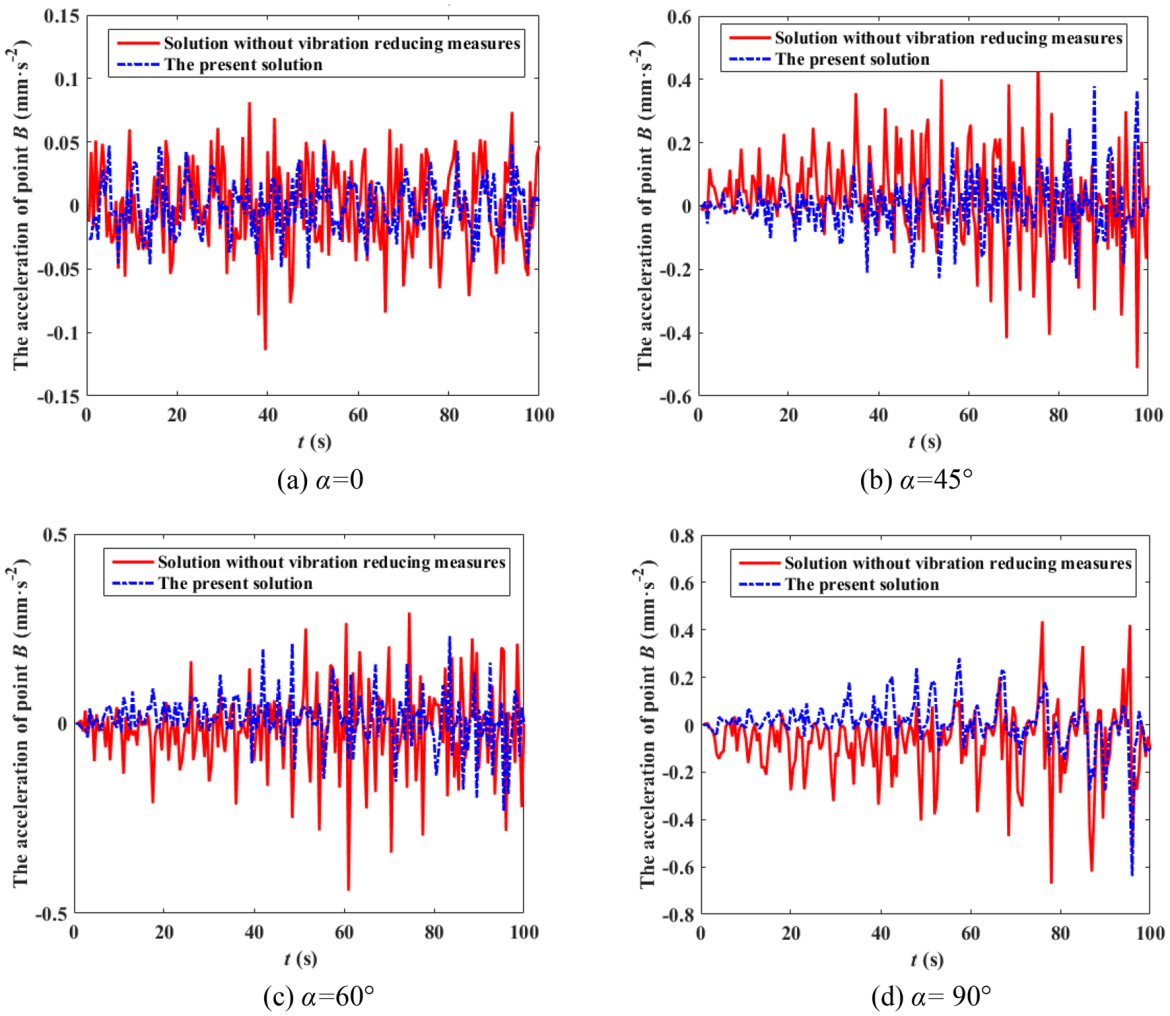
Acceleration time history curve of point *B* under different wind direction.

The boundary flow field of the wind tunnel test is simulated according to roughness landform of class *II*. The height of the wind speed reference point of the wind tunnel is 1.5 m, and the design wind speed of the 500 kV ISLSTT is 42 m/s. Under the condition of the basic wind speed equals to 27 m/s and the wind direction *α* equals to 0, 45°, 60° and 90°, the stress time history curve of root point *A* and the acceleration time history curve of top point *B* are compared with the one without vibration reduction system, which are shown in Figs. 13 and 14 by using dynamic strain gauge to test and analysis the real time data from the sensor of stress as well as acceleration.

It can be seen from Figs. 13 and 14 that compared with the 500 kV ISLSTT without any vibration reduction system, the new vibration reduction system based on wire rope damping structure established in this paper can significantly reduce the maximum stress and acceleration of the transmission tower under the action of wind speed of 27 m/s and wind direction *α* equals to 0, 45°, 60° and 90°, the average value of stress vibration reduction efficiency of root point *A* is 72.88%, 71.46%, 70.39% and 71.83%, respectively. The average value of acceleration vibration reduction efficiency of top point *B* is 51.16% 62.86%, 45.65% and 57.14%, respectively.

The analysis by the wind tunnel test of the 500 kV ISLSTT with the new vibration reduction system also shows that due to the limitation of processing technology, the stress damping efficiency is higher than the one of acceleration.

## Conclusions

In this paper, a new vibration reduction system based on wire rope damping structure is designed. The nonlinear finite element of the 500 kV ISLSTT model with the new vibration reduction system in Jiamusi region is established in ANSYS for dynamic simulation, and the aero-elastic wind tunnel test model is also built to analyze its vibration reduction characteristics, then the time history curves of stress and acceleration at key points under different wind directions are obtained. The specific conclusions are as follows:

1. The response of the 500 kV ISLSTT with the new vibration reduction system to wind vibration is significantly reduced, which shows that the vibration reduction scheme proposed in this paper is feasible and efficient.
2. In the nonlinear finite element model of the 500 kV ISLSTT, the average value of stress damping effect is up to 68.23% and the average value of acceleration damping effect is up to 77.17% in four different wind directions, and the damping effect is consistent in different wind directions.
3. In the aero-elastic wind tunnel test model of the 500 kV ISLSTT, the average value of stress damping effect is up to 72.88% and the average value of acceleration damping effect is up to 62.86% in four different wind directions. However, due to the limitation of the structural processing technology, the damping effect of this new vibration reduction system fluctuates greatly in different wind directions.

## Data Availability

The datasets used and/or analyzed during the current study available from the corresponding author on reasonable request.
